# Novel Resistant Potato Starches on Glycemia and Satiety in Humans

**DOI:** 10.1155/2012/478043

**Published:** 2012-05-13

**Authors:** Mark D. Haub, Julie A. Louk, Tara C. Lopez

**Affiliations:** Food and Metabolism Laboratory, Department of Human Nutrition, Kansas State University, 206 Justin Hall, Manhattan, KS 66506-1400, USA

## Abstract

This study was designed to determine the efficacy of two novel type-four resistant starches (RS4) on postprandial glycemia and ratings of fullness. Volunteers (*n* = 10) completed completed five interventions designed to determine the glycemic and satiety (fullness) effects of the starches (38 g,) alone and when added on top of available carbohydrate. The dose of the starches provided 30 g of resistant starch per treatment. The treatments were: commercial resistant starch added to water (PF−), noncommercial resistant starch added to water (PR−), dextrose solution (DEX, 50 g), and DEX with PenFibe starch (PF+), and DEX with the non-commercial starch added (PR+). Blood glucose was measured in the fasted state and following the randomly assigned treatments at 30, 45, 60, 90, and 120 minutes post-consumption. A visual analog scale was used to determine fullness at each time point. There were no differences in the glucose incremental areas under the curve (iAUC) for PF+ and PR+ compared with DEX. The PF− and PR− treatments had decreased (*P* < 0.05) iAUCs for glucose compared with DEX, PF+, and PR+. There were no treatment differences for RoF. The dose (38 g) of starches did not to alter glucose responses when added on top of 50 g of dextrose.

## 1. Introduction

Diabetes, obesity, and cardiovascular diseases are interrelated, with diet being one factor that links them all together. Specifically, the current obesity epidemic has been suggested to be a result of increased carbohydrate intake [1], while dietary fat has frequently been touted as the primary dietary culprit in the cause of many deleterious metabolic conditions. As a nation, we increased carbohydrate consumption during the latter years of the past century as the obesity epidemic began to surge [1]. While it is laudable to try to change the behavior of individuals so they choose other foods, it might be more effective in the short term to address this nutritional issue by providing bioactive compounds that “behave” like traditional starch yet elicit more favorable metabolic outcomes (acutely and chronically). In other words, let people continue to choose some of the foods they prefer, but make those foods healthier by incorporating bioactive ingredients.

 To that end, incorporating resistant starches into foods by substituting them for the typical starch has been shown to acutely decrease postprandial glucose and insulin [2]. There are five types of resistant starch, with RS types 2, 3, and 4 tending to be studied more frequently. Also, there are varieties of resistant starch within each type [3]. Gram for gram, some resistant starches have been reported to elicit minimal glucose and insulin excursions compared with similar amounts of dextrose [2] and are easily incorporated into regular food items with minimal aversion by consumers [4]. Given the number of resistant starches commercially available, there is a paucity of evidence from human trials illustrating the effects various types of resistant starches have on glucose metabolism. Likewise, some consumers are looking to food to assist them with regulating food intake and/or regulating blood glucose. There is evidence that resistant starch might be able to achieve this by affecting satiety and subsequent food intake, and attenuating postprandial glycemia and insulinemia [2, 5]. To complicate matters, Behall et al. [6] reported that it is imperative that individual starches and fibers be tested for efficacy given the unique structure and function qualities of starches that are quickly becoming available. For example, Haub et al. [7] observed that specific forms of RS2 and RS4 elicit different glycemic responses when compared at the same dose.

 While cross-linked RS4 has been shown to elicit significant reductions in glycemia and insulinemia [2], newer versions are being formulated and might provide even greater and/or different health impacts. One new version of RS4 is commercially available but has not been clinically tested to determine its efficacy to assist with regulating blood glucose and minimizing hyperglycemia. Therefore, the purpose of this study was to determine the glycemic and satiety effects of novel forms of RS4, derived from potato starch. The hypotheses were that consuming the RS4 treatments would not affect glycemia compared with a dextrose-only control solution; and would increase ratings of fullness when added to dextrose control solution.

## 2. Methods

### 2.1. Subjects

The Institutional Review Board of Kansas State University approved the study, and written informed consent was obtained from all volunteers prior to the study. Inclusion criteria were no diagnosis of acute or chronic metabolic and cardiovascular diseases, free of gastrointestinal disorders, body mass index of 20–30 kg/m^2^, and nonsmokers. Ten adults were recruited and completed the study.

### 2.2. Study Design

All trials were performed at the Human Metabolism Laboratory at Kansas State University. Volunteers completed five trials via a single-blind randomized crossover design (we did not alter the water to match the taste/color of the dextrose solution). However, neither the subject nor the technician knew which RS treatment they were receiving. During each visit, volunteers consumed one of the following: dextrose solution of 50 g oral glucose tolerance beverage (DEX), 38 g of PF mixed into DEX (PF+), 38 g of PR mixed into DEX (PR+), 38 g of PF mixed in to water (PF−), and 38 g of PR mixed into water (PR−). The doses of RS were chosen to provide 30 g of resistant starch as 80% of each resistant starch was determined to contain RS and dietary fiber (Table 1). The resistant starches were selected as they are novel, were uniquely produced using proprietary techniques, and had not been clinically tested for efficacy regarding their effects on glycemia as resistant starches do not necessarily elicit the same glycemic response [7]. The treatments were randomly administered in crossover fashion using a Latin square design.

 After providing informed consent, as approved by the University Research Compliance Office at Kansas State University, volunteers were enrolled into the study. For testing, volunteers arrived to the laboratory in a 10-hour fasted state. The fasting capillary blood samples were drawn by finger stick prior to drinking the assigned treatment for that day. Volunteers were allowed 10 minutes to ingest the treatment. The remaining blood samples were collected at 30, 45, 60, 90, and 120 min after ingesting the treatment. Blood samples were analyzed in duplicate for glucose concentration using a YSI 2300 STAT (Yellow Springs, OH).

 After blood was collected, the participants were provided a visual analog scale (Satiety Labeled Intensity Magnitude (SLIM) scale) to provide a rating of fullness (RoF) as used by others [8]. Volunteers were not shown previous surveys before the next treatment. At the conclusion of the study, one technician (blind to treatments) recorded the individual RoF responses.

### 2.3. Statistics

The incremental area under the curve (iAUC) was calculated for glucose using the trapezoid model (Prism v5, GraphPad). A repeated measures analysis of variance was used to determine the presence of significant main and interaction effects, and Bonferroni post-hoc assessments were performed to determine between treatment effects and significance set at *P* = 0.05 (Prism v5, GraphPad).

## 3. Results

### 3.1. Glycemia and Fullness

**Figure 1:**
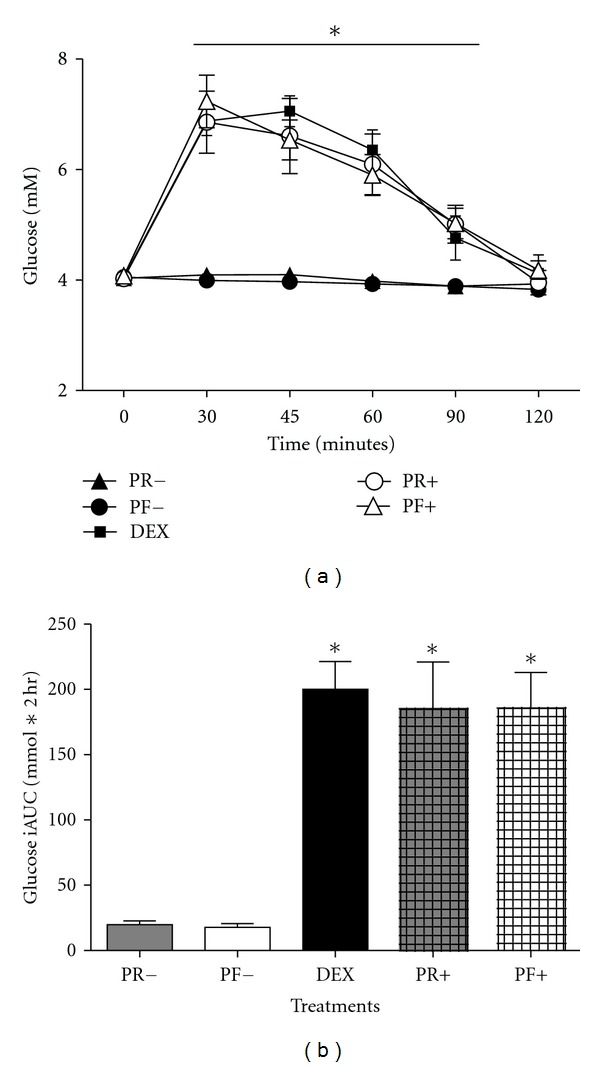
(a) The glucose responses to 38 g of resistant starches with water only (PR− and PF−), with 50 g of dextrose (PR+ and PF+), and 50 g of dextrose only (DEX). * = *P* < 0.05 for differences between PF− and PR− compared with DEX, PF+, and PR+, from 30 minutes to 90 minutes, means ± SD. (b) The iAUC values for each treatment. * = different (*P* < 0.0001) from PF− and PR−, means ± SEM.

**Figure 2:**
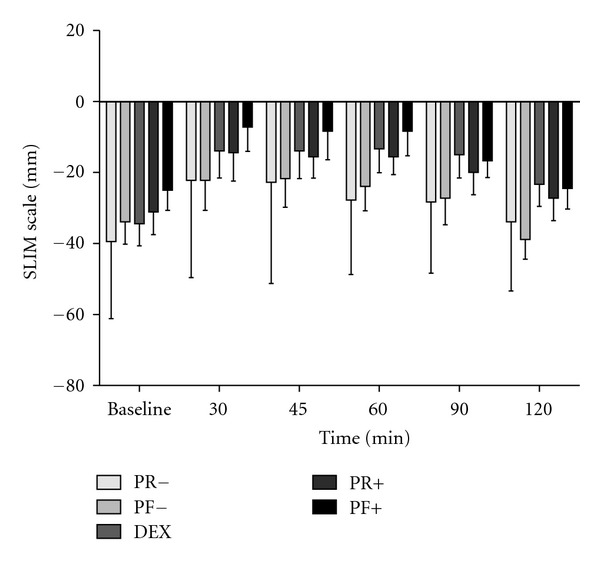
The satiety responses, using the Satiety Labeled Intensity Magnitude scale (SLIM), to consuming 38 g of resistant starches with water, (PF− and PR−), with 50 g of dextrose (PF+ and PR+), and 50 g of dextrose only (DEX), means ± SD.

There was a significant (*P* < 0.05) difference between the two water trials and three dextrose trials, with the water + RS trials eliciting a significantly decreased glycemic response compared with the three dextrose trials (Figure 1). There were no treatment differences (*P* > 0.05) within the three DEX treatments (DEX, PR+, and PF+) or within the two water treatments (PR− and PF−). There was a significant main effect for time in PR+, PF+, and DEX, while there were no time effects following the ingestion of 38 g of each resistant starch mixed with water. The PR+ and PF+ treatments with 38 g of potato-derived modified starches added to DEX did not increase the glycemic response compared with DEX alone. There were no treatment effects for RoF (Figure 2).

## 4. Discussion

This study illustrates that two novel potato-derived resistant starches did not elicit a glycemic response when added to 296 mL of water. Likewise, there was no treatment effect of each RS4 added to DEX when compared to DEX alone. The glycemic results of the present study are similar to data from our previous assessment of a wheat-derived RS4 [7] in that the excursion from baseline was minimal when PF and PR were ingested when added to water. The treatments did not differ in RoF.

 The acute glycemic effect of resistant starch, regardless of type, has been studied extensively and has been shown to decrease glucose and insulin when ingested as part of a food [2, 6, 9], but few studies to date have concomitantly investigated the effects on satiety. A study by Raben et al. [10], who also used potato starch, had volunteers ingest meals containing raw or pregelatinized potato starch on postprandial glucose and insulin concentrations and satiety. They observed that the replacement of raw potato starch (increased RS content) for pregelatinized starch elicited decreased glucose and insulin levels. Contrary to the present observations, they reported that the resistant starch meal elicited a significant (*P* < 0.05) decrease in fullness and satisfaction. The difference between their observations and those of the present study are likely due to the methods of incorporating the resistant starch into the treatments that were ingested. Raben et al. [10] replaced digestible starch with resistant starch, while we added the starch to the digestible carbohydrate. Thus, the treatments used by Raben et al. did not use treatments that had equal amounts of readily digestible starch. Since the resistant starch treatment had 50% less energy from readily digestible starch, their decreased postprandial glucose and insulin responses are expected and are in agreement with the glucose differences observed in the present study between the PF− and PR− treatments when compared with DEX, PF+, and PR+ treatments, as the PF− and PR− treatments did not contain 50 g of dextrose. The difference in satiety results reported between studies may, also, be due to the energy differences of the meals reported by Raben et al. [10], as their RS treatment was reported to contain 367 kJ, while the pregelatinized starch meal contained 917 kJ. However, a study by Leeman et al. [11] supports the satiety responses observed in the present study as they reported no difference in ratings of satiety for potato foods (French fries and boiled potatoes) with differing levels of energy content, indicating that factors other than energy content, such as energy density [12], may need to be taken into account when assessing satiety and subsequent energy intake.

The present data, also, support the notion that postprandial glycemia and satiety responses may not always coincide. Even though subjects were consuming more energy with each dextrose treatment (PF+, PR+, and DEX), the reported satiety responses were not different from those observed with PF− and PR−. This observation has been previously reported [13]. An explanation for the lack of an RoF effect might be that the beverages did not reach a portion threshold to elicit a satiety response [11].

The limitations of this study are as follows: the starches were not incorporated into a food (bread, muffin, etc.), RoF could have been influenced by volume of intake as the PF+ and PR+ treatments (88 g of carbohydrate) had a greater volume than the DEX trial (50 g of carbohydrate), the sample size was powered for glycemia and not fullness, only one outcome of satiety was assessed. Even with shortcomings, this study is significant as it illustrates that glycemia did not influence RoF indicating that beverage-induced increases in blood glucose do not appear to impact RoF. Future studies need to measure intake overtime and take more measures of satiety to better understand how results from acute studies translate to habitual eating.

In conclusion, this study presents novel data regarding the combined physical (glucose) and psychological (satiety) effects of a potato-derived resistant starch. These data indicate that 38 g of PF and PR added to water do not affect capillary glucose over two hours. There was no treatment effect for RoF. Likewise, when 38 g of PF and PR were added on top of 50 g of dextrose (88 g of carbohydrate), those resistant starches did not affect glucose or satiety responses when compared with the DEX (50 g of carbohydrate) treatment. Since the addition of these starches did not affect blood glucose, these RS products might be useful substitutions for standard rapidly digestible starches to decrease postprandial glucose, and likely insulin, to better control blood glucose when incorporated into a food product. However, those foods would need to be clinically tested to insure the effects translate.

## Figures and Tables

**Table 1:** The nutrient composition as provided per 38 g of each ingredient.

	PR	PF
Energy (kJ)		
Total	129.2	129.2
Net	25.8	25.8
Ash (g)	1.6	1.6
Carbohydrates (g)	32.3	32.3
Dietary fiber (g)	30.4	30.4
Resistant starch (g)	30.4	30.4
Sodium (g)	0.3	0.3
Calcium (mg)	16	16
Phosphorous (g)	0.4	0.4
Moisture (g)	14	14

PR = noncommercial resistant starch

PF = commercial resistant starch (PenFibe).
